# Supplementary material for: Carotenoid Biosynthetic Genes in Cabbage: Genome-Wide Identification, Evolution, and Expression Analysis
Source: Genes (Basel). 2021 Dec 20;12(12):2027. doi: 10.3390/genes12122027 (PMC8701174; doi:10.3390/genes12122027)
Supplement: Supplementary file 1 [file genes-12-02027-s001.zip › Fig. S1.pdf]

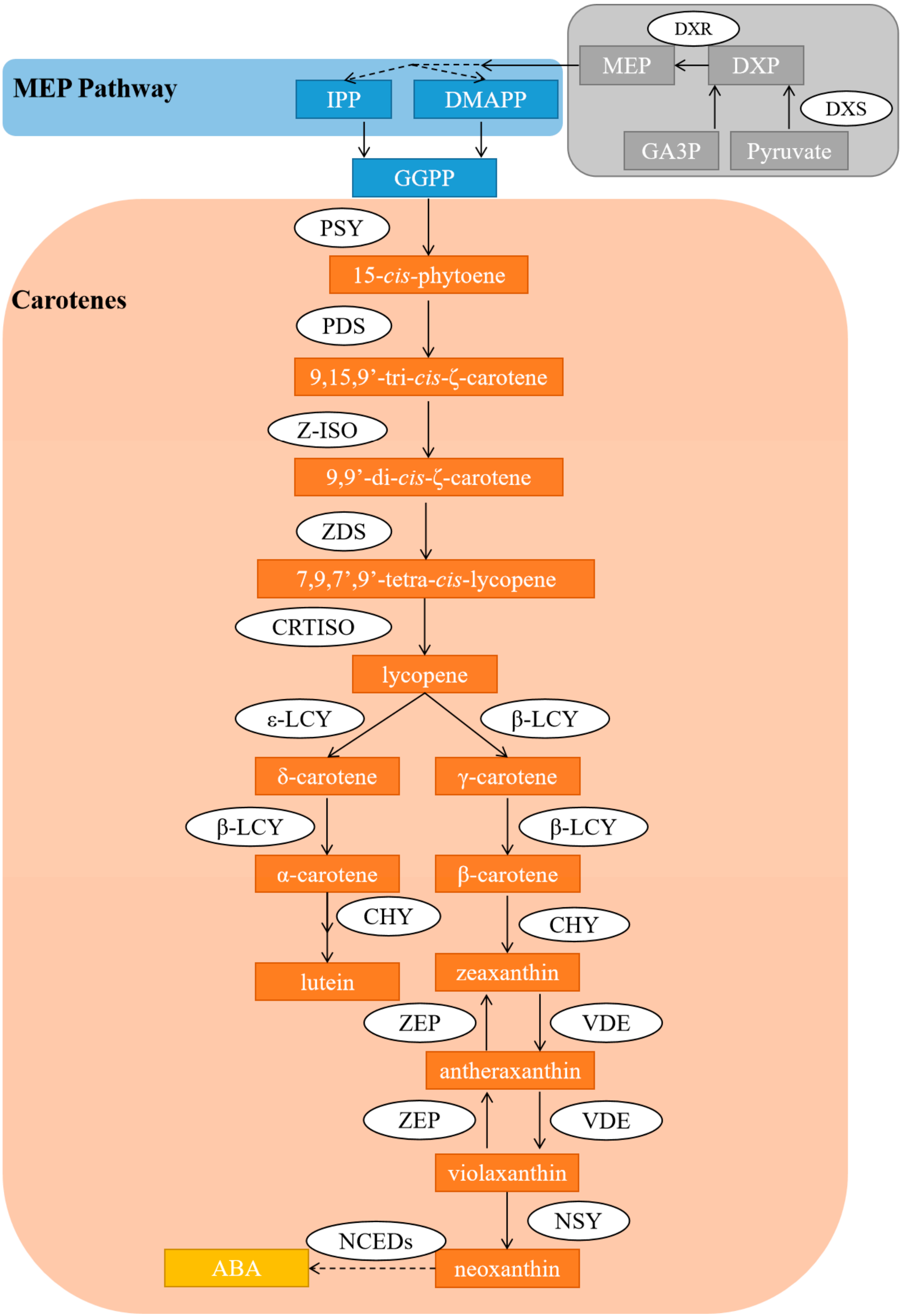

Figure S1. Carotenoid biosynthetic pathway. Abbreviations of enzymes: DXR, deoxy-D-xylulose 5-phosphate reductoisomerase; DXS, deoxy-D-xylulose 5-phosphate synthase; PSY, phytoene synthase; PDS, phytoene desaturase; ZDS, z-carotene desaturase; CRTISO, carotenoid isomerase; β-LCY, lycopene β-cyclase; lycopene ε-LCY, ε-cyclase; CHY, carotene hydroxylase; VDE, violaxanthin de-epoxidase; ZEP, zeaxanthin epoxidase; NSY, neoxanthin synthase; NCEDs, 9-cis-epoxy-carotenoid dioxygenases.
