# Supplementary material for: Carotenoid Biosynthetic Genes in Cabbage: Genome-Wide Identification, Evolution, and Expression Analysis
Source: Genes (Basel). 2021 Dec 20;12(12):2027. doi: 10.3390/genes12122027 (PMC8701174; doi:10.3390/genes12122027)
Supplement: Supplementary file 1 [file genes-12-02027-s001.zip › TableS1.pdf]

Table S1. The primer sequences information used in this study

Primer name	Forward (5′-3′)	Reverse (5′-3′)
qP-Actin	CCAGAGGTCTTGTTCCAGCCATC	GTTCCACCACTGAGCACAATGTTAC
qP-BoPSY.1	TCCTCTCTAATGAGCTACAGAAGGA	ATCGATGCCAGGAGAAGTAGACCTG
qP-BoPDS.1	TTGAAGAATGGATGAGAAAGCAGGG	CATCTTCGAACCATGTTTCTCCTGA
qP-BoZDS	TTTAACGCCAGGAGATCCCTAC	TATGAACCAGCGAGGAAGAAGT
qP-BoLYC	AAACTCATTTGGCCTAACAACTAC	CAGTGACTTTAGCCTGATGGAAT
	CGATCGTCTCACAACATGTCTTCTGT	CGATGGTCTCACTCTGAGTATGTTGG
PSY1-1	AGCAGTGTTATGGGTTGCTTCCTCTTC	TAAGCTGATTGGCTATACCGAGAGC
	TCCTAA	CAAGGCAG
	CGATCGTCTCAAGAGACGTTGGCGA	CAGTCGTCTCATACAAGTTGTTCCTC
PSY1-2	AGATGCGAGAAGAGGAAGAGTTTAT	TTGAACTTGGAGTCTTTAGTACTGAT
	CTGCCCCAA	TTAGCAT
	CGATCGTCTCACAACATGTCTTCTGT	CAGTCGTCTCATACTTAACTTGAAA
PSY2	AGCAGTGTTATGGGTTGCTGCTTCTTC	CCTTTACTACTGATTTAGCATAAGCT
	TCCAA	AATGGAAGAG
	CGATCGTCTCACAACATGTCTTCTGT	CAGTCGTCTCATACTCATCTTGAACT
PSY3	AGCAGTGTTATGGGTTGCTCCCTCTTC	TGAAGCCTTTAGTATTGATTTAGCAT
	TCCAA	AAGCTAATG
