# Supplementary material for: Carotenoid Biosynthetic Genes in Cabbage: Genome-Wide Identification, Evolution, and Expression Analysis
Source: Genes (Basel). 2021 Dec 20;12(12):2027. doi: 10.3390/genes12122027 (PMC8701174; doi:10.3390/genes12122027)
Supplement: Supplementary file 1 [file genes-12-02027-s001.zip › TableS2.pdf]

Table S2. The accession No. of amino acid sequences of *PSY* in 23 kinds of plants

Plant Species	The Latin name	Amino NO.
Tomato	*Solanum lycopersicum*	ACE75885.1
Chilli	*Capsicum annuum*	ACE78189.1
Medlar	*Lycium barbarum*	AAW88383.1
Sweet potato	*Ipomoea batatas*	BAI47572.1
Cape jasmine	*Gardenia jasminoides*	AEF59491.1
Osmanthus	*Osmanthus fragrans*	AFK66771.1
Agastache	*Pogostemon cablin*	AHJ90431.1
Persimmon	*Diospyros kaki*	ACM44688.1
Kiwifruit	*Actinidia deliciosa*	ACO53104.1
Watermelon	*Citrullus lanatus*	AGT57744.1
Pumpkin	*Cucurbita moschata*	AEK86564.1
Papaya	*Carica papaya*	ABG72805.1
Arabidopsis	*Arabidopsis thaliala*	AED92401.1
Narcissus	*Narcissus tazetta*	ABI98829.1
Lilium brownii	*Lilium lancifolium*	ADW08475.1
Alfalfa	*Medicago sativa*	AIT98180.1
Carrot	*Daucus carota*	Q9SSU8.1
Strawberry	*Fragaria × ananassa*	ACR61392.1
Apple	*Malus domestica*	AKU36798.1
Rice	*Oryza sativa Indica*	AAS18307.1
Maize	*Zea mays*	AAX13806.1
Sorghum	*Sorghum bicolor*	AAW28996.1
Haematococcus	*Haematococcus lacustris*	AAY53806.1
